# Dichlorido(2-meth­oxy-1,10-phenanthroline-κ^2^ *N*,*N*′)zinc(II)

**DOI:** 10.1107/S160053680801180X

**Published:** 2008-05-03

**Authors:** Hong Li, Tai Qiu Hu, Shi Guo Zhang

**Affiliations:** a Department of Chemistry and Chemical Engineering, Institute of Materials Chemistry, Binzhou University, Binzhou 256603, People’s Republic of China; b Department of Chemistry, Shandong Normal University, Jinan 250014, People’s Republic of China

## Abstract

There are two molecules of the title complex, [ZnCl_2_(C_13_H_10_N_2_O)], in the asymmetric unit. Each Zn atom assumes a distorted tetra­hedral ZnN_2_Cl_2_ coordination geometry. There are weak π–π stacking inter­actions between adjacent 1,10-phenanthroline rings [centroid–centroid distances = 3.6356 (18) and 3.6353 (18) Å].

## Related literature

For a related structure, see: Zheng *et al.* (2003 ▶).
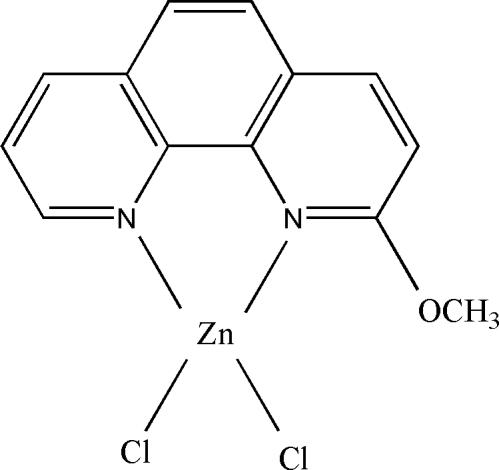


## Experimental

### 

#### Crystal data

[ZnCl_2_(C_13_H_10_N_2_O)]
*M* *_r_* = 346.50
Triclinic, 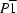

*a* = 9.9051 (17) Å
*b* = 11.5654 (19) Å
*c* = 12.774 (2) Å
α = 91.849 (2)°
β = 108.295 (2)°
γ = 98.672 (2)°
*V* = 1368.5 (4) Å^3^
*Z* = 4
Mo *K*α radiation
μ = 2.18 mm^−1^
*T* = 173 (2) K
0.51 × 0.40 × 0.10 mm

#### Data collection

Bruker SMART APEX CCD diffractometer
Absorption correction: multi-scan (*SADABS*; Sheldrick, 1996 ▶) *T*_min_ = 0.403, *T*_max_ = 0.812
7507 measured reflections
5264 independent reflections
4386 reflections with *I* > 2σ(*I*)
*R*_int_ = 0.019

#### Refinement

*R*[*F* ^2^ > 2σ(*F* ^2^)] = 0.034
*wR*(*F* ^2^) = 0.092
*S* = 1.05
5264 reflections
345 parameters
H-atom parameters constrained
Δρ_max_ = 0.59 e Å^−3^
Δρ_min_ = −0.35 e Å^−3^

### 

Data collection: *SMART* (Bruker, 1997 ▶); cell refinement: *SAINT* (Bruker, 1997 ▶); data reduction: *SAINT*; program(s) used to solve structure: *SHELXTL* (Sheldrick, 2008 ▶); program(s) used to refine structure: *SHELXTL*; molecular graphics: *SHELXTL*; software used to prepare material for publication: *SHELXTL*.

## Supplementary Material

Crystal structure: contains datablocks I, global. DOI: 10.1107/S160053680801180X/ww2117sup1.cif

Structure factors: contains datablocks I. DOI: 10.1107/S160053680801180X/ww2117Isup2.hkl

Additional supplementary materials:  crystallographic information; 3D view; checkCIF report

## Figures and Tables

**Table:** 

Cl1—Zn2	2.1911 (8)
Cl2—Zn2	2.2139 (9)
Cl3—Zn1	2.1949 (8)
Cl4—Zn1	2.2315 (8)
N1—Zn1	2.054 (2)
N2—Zn1	2.076 (2)
N3—Zn2	2.098 (2)
N4—Zn2	2.056 (2)

**Table:** 

N1—Zn1—N2	81.15 (8)
N1—Zn1—Cl3	119.22 (6)
N2—Zn1—Cl3	114.55 (7)
N1—Zn1—Cl4	113.92 (6)
N2—Zn1—Cl4	107.63 (6)
Cl3—Zn1—Cl4	115.13 (3)
N4—Zn2—N3	80.57 (9)
N4—Zn2—Cl1	116.23 (7)
N3—Zn2—Cl1	117.56 (7)
N4—Zn2—Cl2	116.69 (7)
N3—Zn2—Cl2	104.48 (7)
Cl1—Zn2—Cl2	115.82 (4)

**Fig. 1.**
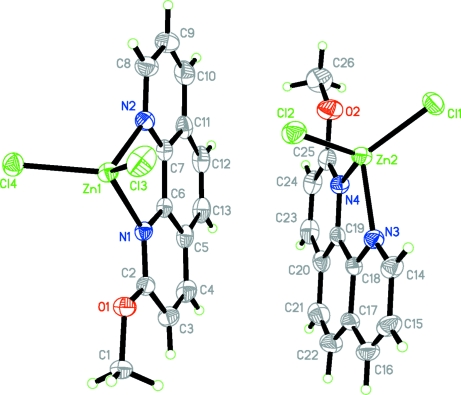
View of complex (I), showing the the atom numbering scheme with thermal ellipsoids drawn at the 30% probability level.

## supplementary crystallographic information

### Comment

The derivatives of 1,10-phenanthroline play a pivotal role in the area of modern coordination chemistry (Zheng *et al.*, 2003), but the complexes with 2-methoxy-1,10-phenanthroline as ligand has not been reported. We are interested in this kind of ligands and here we report a new complex (I) with 2-methoxy-1,10-phenanthroline as ligand.

The coordination structure of (I) is shown in Fig. 1. Each Zn(II) ion is coordinated with two Cl ions and two N atoms, and Zn—Cl bond lengths range from 2.1911 (8)Å to 2.2315 (8) Å and Zn—N bond lengths vary from 2.054 (2) Å to 2.098 (2)Å. Obviously Zn1 and Zn2 atoms are in a distorted tetrahedral geometry (Table 1). There are weak π-π stacking interaction between neighbouring 1,10-phenanthroline ligands. The centroid-to-centroid distances and the centroid-to-plane distances are *Cg*1···*Cg*2^i^ = 3.6356 (18)Å and *Cg*1···*Cg*2^i^_perp_ = 3.476 Å; *Cg*3···*Cg*4^ii^ = 3.6353 (18)Å and *Cg*3···*Cg*4^i^_perp_ = 3.434Å, and Cgi···Cgj should be the centroid-to-centroid distance while the Cgi···Cgj_prep_ should be the centroid-to-plane distance. *Cg*1, *Cg*2, *Cg*3 and *Cg*4 are the centroids of the rings N3/C14-C18, C17-C22, N2/C7-C11 and C5-C7/C11-C13, respectively. [symmetry codes: (i) 2-X, 2-Y, 2-Z; (ii) 1-X,1-Y,1-Z]

### Experimental

A methanol solution (10 ml) of ZnCl_2_ (0.0785 g, 0.576 mmol) was added into 10 ml of methanol solution containing 2-methoxy-1,10-phenanthroline (0.1205 g, 0.573 mmol), and the mixed solution was stirred for a few minutes. Colorless crystals were obtained after the solution had been allowed to stand at room temperature for one week.

### Refinement

H atoms were placed in calculated positions (C—H = 0.96 Å for methyl group and C—H = 0.93 Å for phenyl H atoms) and refined as riding with *U*_iso_ = 1.5 *U*_eq_(C) for methyl H and *U*_iso_ = 1.2 *U*_eq_(C) for phenyl H.

### Crystal data

**Table:**

[ZnCl_2_(C_13_H_10_N_2_O)]	*Z* = 4
*M**_r_* = 346.50	*F*_000_ = 696
Triclinic, *P*1	*D*_x_ = 1.682 Mg m^−^^3^
Hall symbol: -P 1	Mo *K*α radiation λ = 0.71073 Å
*a* = 9.9051 (17) Å	Cell parameters from 3641 reflections
*b* = 11.5654 (19) Å	θ = 2.4–27.0º
*c* = 12.774 (2) Å	µ = 2.18 mm^−^^1^
α = 91.849 (2)º	*T* = 173 (2) K
β = 108.295 (2)º	Block, colorless
γ = 98.672 (2)º	0.51 × 0.40 × 0.10 mm
*V* = 1368.5 (4) Å^3^	

### Data collection

**Table:**

Bruker SMART APEX CCD diffractometer	5264 independent reflections
Radiation source: fine-focus sealed tube	4386 reflections with *I* > 2σ(*I*)
Monochromator: graphite	*R*_int_ = 0.019
*T* = 446(2) K	θ_max_ = 26.0º
φ and ω scans	θ_min_ = 1.7º
Absorption correction: multi-scan(SADABS; Sheldrick, 1996)	*h* = −12→10
*T*_min_ = 0.403, *T*_max_ = 0.812	*k* = −14→12
7507 measured reflections	*l* = −15→14

### Refinement

**Table:**

Refinement on *F*^2^	Secondary atom site location: difference Fourier map
Least-squares matrix: full	Hydrogen site location: inferred from neighbouring sites
*R*[*F*^2^ > 2σ(*F*^2^)] = 0.034	H-atom parameters constrained
*wR*(*F*^2^) = 0.092	*w* = 1/[σ^2^(*F*_o_^2^) + (0.0513*P*)^2^] where *P* = (*F*_o_^2^ + 2*F*_c_^2^)/3
*S* = 1.05	(Δ/σ)_max_ = 0.001
5264 reflections	Δρ_max_ = 0.59 e Å^−^^3^
345 parameters	Δρ_min_ = −0.35 e Å^−^^3^
Primary atom site location: structure-invariant direct methods	Extinction correction: none

### Special details

**Table:**

Geometry. All e.s.d.'s (except the e.s.d. in the dihedral angle between two l.s. planes) are estimated using the full covariance matrix. The cell e.s.d.'s are taken into account individually in the estimation of e.s.d.'s in distances, angles and torsion angles; correlations between e.s.d.'s in cell parameters are only used when they are defined by crystal symmetry. An approximate (isotropic) treatment of cell e.s.d.'s is used for estimating e.s.d.'s involving l.s. planes.
Refinement. Refinement of *F*^2^ against ALL reflections. The weighted *R*-factor *wR* and goodness of fit *S* are based on *F*^2^, conventional *R*-factors *R* are based on *F*, with *F* set to zero for negative *F*^2^. The threshold expression of *F*^2^ > σ(*F*^2^) is used only for calculating *R*-factors(gt) *etc*. and is not relevant to the choice of reflections for refinement. *R*-factors based on *F*^2^ are statistically about twice as large as those based on *F*, and *R*- factors based on ALL data will be even larger.

### Fractional atomic coordinates and isotropic or equivalent isotropic displacement parameters (Å^2^)

**Table:**

	*x*	*y*	*z*	*U*_iso_*/*U*_eq_	
C1	0.5226 (3)	0.3267 (3)	1.0172 (2)	0.0481 (7)	
H1A	0.5587	0.2596	0.9974	0.072*	
H1B	0.4565	0.3028	1.0567	0.072*	
H1C	0.6016	0.3835	1.0634	0.072*	
C2	0.5263 (3)	0.4341 (2)	0.8608 (2)	0.0368 (6)	
C3	0.6781 (3)	0.4562 (2)	0.8936 (2)	0.0462 (7)	
H3	0.7326	0.4304	0.9593	0.055*	
C4	0.7431 (3)	0.5151 (3)	0.8286 (3)	0.0505 (7)	
H4	0.8433	0.5282	0.8487	0.061*	
C5	0.6615 (3)	0.5573 (2)	0.7302 (2)	0.0429 (6)	
C6	0.5121 (3)	0.5345 (2)	0.7051 (2)	0.0334 (5)	
C7	0.4210 (3)	0.5801 (2)	0.6104 (2)	0.0370 (6)	
C8	0.1928 (4)	0.6050 (3)	0.5073 (2)	0.0518 (7)	
H8	0.0938	0.5918	0.4950	0.062*	
C9	0.2462 (4)	0.6718 (3)	0.4356 (3)	0.0622 (9)	
H9	0.1838	0.7031	0.3772	0.075*	
C10	0.3897 (4)	0.6909 (3)	0.4518 (2)	0.0581 (9)	
H10	0.4259	0.7341	0.4034	0.070*	
C11	0.4844 (3)	0.6458 (2)	0.5413 (2)	0.0453 (7)	
C12	0.6372 (4)	0.6614 (3)	0.5662 (3)	0.0540 (8)	
H12	0.6791	0.7013	0.5191	0.065*	
C13	0.7222 (3)	0.6196 (3)	0.6564 (3)	0.0518 (8)	
H13	0.8215	0.6315	0.6707	0.062*	
C14	0.7048 (3)	0.9284 (3)	1.0543 (2)	0.0493 (7)	
H14	0.6289	0.9633	1.0609	0.059*	
C15	0.7847 (4)	0.8771 (3)	1.1450 (2)	0.0577 (8)	
H15	0.7612	0.8760	1.2100	0.069*	
C16	0.8981 (4)	0.8284 (3)	1.1366 (2)	0.0593 (9)	
H16	0.9532	0.7941	1.1966	0.071*	
C17	0.9327 (3)	0.8296 (2)	1.0379 (2)	0.0463 (7)	
C18	0.8429 (3)	0.8819 (2)	0.9508 (2)	0.0396 (6)	
C19	0.8719 (3)	0.8864 (2)	0.8467 (2)	0.0388 (6)	
C20	0.9915 (3)	0.8434 (2)	0.8365 (3)	0.0474 (7)	
C21	1.0808 (4)	0.7921 (3)	0.9269 (3)	0.0642 (9)	
H21	1.1610	0.7640	0.9198	0.077*	
C22	1.0512 (4)	0.7837 (3)	1.0217 (3)	0.0628 (9)	
H22	1.1092	0.7472	1.0784	0.075*	
C23	1.0158 (4)	0.8553 (3)	0.7330 (3)	0.0594 (9)	
H23	1.0949	0.8294	0.7215	0.071*	
C24	0.9236 (3)	0.9045 (3)	0.6509 (3)	0.0526 (8)	
H24	0.9398	0.9134	0.5834	0.063*	
C25	0.8042 (3)	0.9416 (3)	0.6693 (2)	0.0486 (7)	
C26	0.7173 (5)	1.0057 (4)	0.4866 (3)	0.0829 (13)	
H26A	0.8048	1.0594	0.4954	0.124*	
H26B	0.6365	1.0377	0.4417	0.124*	
H26C	0.7209	0.9322	0.4514	0.124*	
Cl1	0.57382 (10)	1.15815 (7)	0.79281 (6)	0.0581 (2)	
Cl2	0.40833 (9)	0.84139 (7)	0.75709 (6)	0.0574 (2)	
Cl3	0.09774 (8)	0.51212 (8)	0.79552 (7)	0.0641 (2)	
Cl4	0.12965 (8)	0.27076 (6)	0.60771 (5)	0.04690 (18)	
N1	0.4460 (2)	0.47186 (17)	0.76905 (16)	0.0327 (5)	
N2	0.2774 (2)	0.55965 (19)	0.59217 (17)	0.0393 (5)	
N3	0.7313 (3)	0.93004 (19)	0.95981 (17)	0.0410 (5)	
N4	0.7788 (3)	0.93342 (19)	0.76405 (17)	0.0398 (5)	
O1	0.4488 (2)	0.37827 (16)	0.91786 (14)	0.0420 (4)	
O2	0.7019 (3)	0.9874 (2)	0.59364 (16)	0.0640 (6)	
Zn1	0.22524 (3)	0.44580 (3)	0.70131 (2)	0.03586 (10)	
Zn2	0.60675 (3)	0.97534 (3)	0.80582 (2)	0.03979 (11)	

### Atomic displacement parameters (Å^2^)

**Table:**

	*U*^11^	*U*^22^	*U*^33^	*U*^12^	*U*^13^	*U*^23^
C1	0.060 (2)	0.0456 (17)	0.0352 (14)	0.0128 (14)	0.0073 (14)	0.0087 (12)
C2	0.0341 (14)	0.0349 (14)	0.0394 (14)	0.0070 (11)	0.0090 (12)	0.0005 (11)
C3	0.0316 (15)	0.0500 (17)	0.0519 (16)	0.0070 (13)	0.0066 (13)	0.0025 (14)
C4	0.0281 (15)	0.0530 (18)	0.0653 (19)	0.0035 (13)	0.0100 (14)	−0.0037 (15)
C5	0.0353 (15)	0.0393 (15)	0.0547 (16)	−0.0019 (12)	0.0205 (13)	−0.0073 (13)
C6	0.0312 (13)	0.0297 (13)	0.0395 (13)	0.0017 (10)	0.0141 (11)	−0.0043 (10)
C7	0.0424 (16)	0.0308 (13)	0.0391 (14)	0.0000 (11)	0.0186 (12)	−0.0025 (11)
C8	0.0515 (19)	0.0526 (18)	0.0488 (17)	0.0105 (15)	0.0111 (15)	0.0138 (14)
C9	0.079 (3)	0.059 (2)	0.0433 (17)	0.0102 (18)	0.0118 (17)	0.0194 (15)
C10	0.085 (3)	0.0454 (18)	0.0474 (17)	−0.0003 (17)	0.0303 (18)	0.0102 (14)
C11	0.0601 (19)	0.0333 (15)	0.0448 (15)	−0.0037 (13)	0.0258 (15)	−0.0006 (12)
C12	0.066 (2)	0.0428 (17)	0.0623 (19)	−0.0104 (15)	0.0430 (18)	−0.0056 (15)
C13	0.0414 (17)	0.0467 (17)	0.069 (2)	−0.0087 (13)	0.0290 (16)	−0.0081 (15)
C14	0.0555 (19)	0.0522 (18)	0.0388 (15)	0.0018 (14)	0.0164 (14)	0.0057 (13)
C15	0.067 (2)	0.062 (2)	0.0357 (15)	−0.0041 (17)	0.0111 (15)	0.0122 (14)
C16	0.064 (2)	0.0517 (19)	0.0446 (17)	−0.0045 (16)	−0.0010 (16)	0.0167 (14)
C17	0.0420 (17)	0.0342 (15)	0.0501 (16)	0.0008 (12)	−0.0008 (13)	0.0087 (13)
C18	0.0391 (15)	0.0284 (13)	0.0437 (15)	−0.0002 (11)	0.0054 (12)	0.0047 (11)
C19	0.0376 (15)	0.0285 (13)	0.0459 (15)	−0.0003 (11)	0.0105 (12)	−0.0006 (11)
C20	0.0379 (16)	0.0386 (15)	0.0631 (18)	0.0010 (12)	0.0155 (14)	−0.0026 (14)
C21	0.0428 (19)	0.057 (2)	0.089 (3)	0.0133 (15)	0.0128 (18)	0.0051 (19)
C22	0.050 (2)	0.055 (2)	0.071 (2)	0.0121 (16)	−0.0005 (17)	0.0122 (17)
C23	0.0453 (19)	0.055 (2)	0.081 (2)	0.0036 (15)	0.0288 (18)	−0.0119 (17)
C24	0.0534 (19)	0.0561 (19)	0.0530 (18)	−0.0024 (15)	0.0305 (16)	−0.0081 (15)
C25	0.0572 (19)	0.0431 (16)	0.0443 (16)	−0.0002 (14)	0.0191 (15)	−0.0010 (13)
C26	0.129 (4)	0.091 (3)	0.0415 (18)	0.026 (3)	0.040 (2)	0.0185 (18)
Cl1	0.0790 (6)	0.0437 (4)	0.0556 (4)	0.0216 (4)	0.0216 (4)	0.0094 (3)
Cl2	0.0496 (5)	0.0599 (5)	0.0557 (4)	0.0008 (4)	0.0103 (4)	0.0108 (4)
Cl3	0.0402 (4)	0.0903 (6)	0.0655 (5)	0.0121 (4)	0.0239 (4)	−0.0136 (4)
Cl4	0.0517 (4)	0.0443 (4)	0.0428 (4)	0.0004 (3)	0.0164 (3)	0.0023 (3)
N1	0.0269 (11)	0.0334 (11)	0.0367 (11)	0.0025 (9)	0.0100 (9)	0.0011 (9)
N2	0.0413 (13)	0.0374 (12)	0.0385 (12)	0.0046 (10)	0.0126 (10)	0.0066 (10)
N3	0.0437 (14)	0.0403 (13)	0.0373 (12)	0.0046 (10)	0.0118 (10)	0.0066 (10)
N4	0.0437 (13)	0.0415 (13)	0.0355 (11)	0.0063 (10)	0.0152 (10)	0.0038 (10)
O1	0.0366 (11)	0.0471 (11)	0.0419 (10)	0.0072 (8)	0.0113 (9)	0.0116 (9)
O2	0.0804 (17)	0.0825 (16)	0.0392 (11)	0.0287 (13)	0.0258 (11)	0.0171 (11)
Zn1	0.02763 (17)	0.04296 (19)	0.03691 (17)	0.00325 (13)	0.01139 (13)	0.00511 (13)
Zn2	0.0424 (2)	0.04270 (19)	0.03666 (18)	0.01284 (14)	0.01315 (15)	0.00795 (14)

### Geometric parameters (Å, °)

**Table:**

C1—O1	1.449 (3)	C15—H15	0.9300
C1—H1A	0.9600	C16—C17	1.406 (4)
C1—H1B	0.9600	C16—H16	0.9300
C1—H1C	0.9600	C17—C18	1.407 (4)
C2—N1	1.323 (3)	C17—C22	1.427 (5)
C2—O1	1.330 (3)	C18—N3	1.344 (4)
C2—C3	1.409 (4)	C18—C19	1.447 (4)
C3—C4	1.345 (4)	C19—N4	1.356 (3)
C3—H3	0.9300	C19—C20	1.392 (4)
C4—C5	1.411 (4)	C20—C23	1.423 (4)
C4—H4	0.9300	C20—C21	1.424 (4)
C5—C6	1.393 (4)	C21—C22	1.336 (5)
C5—C13	1.428 (4)	C21—H21	0.9300
C6—N1	1.361 (3)	C22—H22	0.9300
C6—C7	1.434 (4)	C23—C24	1.361 (5)
C7—N2	1.349 (3)	C23—H23	0.9300
C7—C11	1.412 (4)	C24—C25	1.400 (4)
C8—N2	1.323 (3)	C24—H24	0.9300
C8—C9	1.394 (4)	C25—N4	1.315 (3)
C8—H8	0.9300	C25—O2	1.349 (4)
C9—C10	1.353 (5)	C26—O2	1.441 (3)
C9—H9	0.9300	C26—H26A	0.9600
C10—C11	1.404 (4)	C26—H26B	0.9600
C10—H10	0.9300	C26—H26C	0.9600
C11—C12	1.427 (4)	Cl1—Zn2	2.1911 (8)
C12—C13	1.349 (5)	Cl2—Zn2	2.2139 (9)
C12—H12	0.9300	Cl3—Zn1	2.1949 (8)
C13—H13	0.9300	Cl4—Zn1	2.2315 (8)
C14—N3	1.313 (3)	N1—Zn1	2.054 (2)
C14—C15	1.389 (4)	N2—Zn1	2.076 (2)
C14—H14	0.9300	N3—Zn2	2.098 (2)
C15—C16	1.361 (5)	N4—Zn2	2.056 (2)
			
O1—C1—H1A	109.5	N3—C18—C19	117.8 (2)
O1—C1—H1B	109.5	C17—C18—C19	119.2 (3)
H1A—C1—H1B	109.5	N4—C19—C20	123.9 (3)
O1—C1—H1C	109.5	N4—C19—C18	116.8 (2)
H1A—C1—H1C	109.5	C20—C19—C18	119.3 (3)
H1B—C1—H1C	109.5	C19—C20—C23	115.8 (3)
N1—C2—O1	113.0 (2)	C19—C20—C21	119.9 (3)
N1—C2—C3	121.8 (2)	C23—C20—C21	124.3 (3)
O1—C2—C3	125.1 (2)	C22—C21—C20	121.0 (3)
C4—C3—C2	119.0 (3)	C22—C21—H21	119.5
C4—C3—H3	120.5	C20—C21—H21	119.5
C2—C3—H3	120.5	C21—C22—C17	121.5 (3)
C3—C4—C5	120.9 (3)	C21—C22—H22	119.3
C3—C4—H4	119.5	C17—C22—H22	119.3
C5—C4—H4	119.5	C24—C23—C20	120.2 (3)
C6—C5—C4	116.5 (3)	C24—C23—H23	119.9
C6—C5—C13	119.2 (3)	C20—C23—H23	119.9
C4—C5—C13	124.3 (3)	C23—C24—C25	119.1 (3)
N1—C6—C5	122.6 (2)	C23—C24—H24	120.5
N1—C6—C7	117.0 (2)	C25—C24—H24	120.5
C5—C6—C7	120.3 (2)	N4—C25—O2	112.3 (3)
N2—C7—C11	122.8 (3)	N4—C25—C24	122.7 (3)
N2—C7—C6	118.0 (2)	O2—C25—C24	125.0 (3)
C11—C7—C6	119.2 (3)	O2—C26—H26A	109.5
N2—C8—C9	122.5 (3)	O2—C26—H26B	109.5
N2—C8—H8	118.7	H26A—C26—H26B	109.5
C9—C8—H8	118.7	O2—C26—H26C	109.5
C10—C9—C8	119.4 (3)	H26A—C26—H26C	109.5
C10—C9—H9	120.3	H26B—C26—H26C	109.5
C8—C9—H9	120.3	C2—N1—C6	119.0 (2)
C9—C10—C11	120.3 (3)	C2—N1—Zn1	128.98 (18)
C9—C10—H10	119.8	C6—N1—Zn1	111.96 (16)
C11—C10—H10	119.8	C8—N2—C7	118.5 (2)
C10—C11—C7	116.4 (3)	C8—N2—Zn1	130.2 (2)
C10—C11—C12	124.8 (3)	C7—N2—Zn1	111.08 (17)
C7—C11—C12	118.8 (3)	C14—N3—C18	118.5 (2)
C13—C12—C11	121.6 (3)	C14—N3—Zn2	129.7 (2)
C13—C12—H12	119.2	C18—N3—Zn2	111.10 (17)
C11—C12—H12	119.2	C25—N4—C19	118.4 (3)
C12—C13—C5	120.8 (3)	C25—N4—Zn2	128.8 (2)
C12—C13—H13	119.6	C19—N4—Zn2	112.72 (17)
C5—C13—H13	119.6	C2—O1—C1	118.9 (2)
N3—C14—C15	123.2 (3)	C25—O2—C26	118.9 (3)
N3—C14—H14	118.4	N1—Zn1—N2	81.15 (8)
C15—C14—H14	118.4	N1—Zn1—Cl3	119.22 (6)
C16—C15—C14	118.5 (3)	N2—Zn1—Cl3	114.55 (7)
C16—C15—H15	120.7	N1—Zn1—Cl4	113.92 (6)
C14—C15—H15	120.7	N2—Zn1—Cl4	107.63 (6)
C15—C16—C17	120.5 (3)	Cl3—Zn1—Cl4	115.13 (3)
C15—C16—H16	119.7	N4—Zn2—N3	80.57 (9)
C17—C16—H16	119.7	N4—Zn2—Cl1	116.23 (7)
C16—C17—C18	116.1 (3)	N3—Zn2—Cl1	117.56 (7)
C16—C17—C22	124.9 (3)	N4—Zn2—Cl2	116.69 (7)
C18—C17—C22	119.1 (3)	N3—Zn2—Cl2	104.48 (7)
N3—C18—C17	123.0 (3)	Cl1—Zn2—Cl2	115.82 (4)
			
N1—C2—C3—C4	−1.5 (4)	O1—C2—N1—Zn1	−4.6 (3)
O1—C2—C3—C4	−179.2 (3)	C3—C2—N1—Zn1	177.47 (19)
C2—C3—C4—C5	1.7 (4)	C5—C6—N1—C2	2.3 (4)
C3—C4—C5—C6	0.0 (4)	C7—C6—N1—C2	−176.0 (2)
C3—C4—C5—C13	−179.4 (3)	C5—C6—N1—Zn1	−175.99 (19)
C4—C5—C6—N1	−2.0 (4)	C7—C6—N1—Zn1	5.7 (3)
C13—C5—C6—N1	177.4 (2)	C9—C8—N2—C7	0.7 (4)
C4—C5—C6—C7	176.2 (2)	C9—C8—N2—Zn1	−173.7 (2)
C13—C5—C6—C7	−4.3 (4)	C11—C7—N2—C8	−1.4 (4)
N1—C6—C7—N2	1.0 (3)	C6—C7—N2—C8	177.6 (2)
C5—C6—C7—N2	−177.3 (2)	C11—C7—N2—Zn1	174.0 (2)
N1—C6—C7—C11	−180.0 (2)	C6—C7—N2—Zn1	−7.1 (3)
C5—C6—C7—C11	1.6 (4)	C15—C14—N3—C18	−1.4 (4)
N2—C8—C9—C10	0.6 (5)	C15—C14—N3—Zn2	168.3 (2)
C8—C9—C10—C11	−1.3 (5)	C17—C18—N3—C14	0.1 (4)
C9—C10—C11—C7	0.6 (4)	C19—C18—N3—C14	−179.0 (2)
C9—C10—C11—C12	−179.6 (3)	C17—C18—N3—Zn2	−171.4 (2)
N2—C7—C11—C10	0.7 (4)	C19—C18—N3—Zn2	9.5 (3)
C6—C7—C11—C10	−178.2 (2)	O2—C25—N4—C19	178.6 (2)
N2—C7—C11—C12	−179.1 (2)	C24—C25—N4—C19	−0.5 (4)
C6—C7—C11—C12	2.0 (4)	O2—C25—N4—Zn2	2.1 (4)
C10—C11—C12—C13	177.2 (3)	C24—C25—N4—Zn2	−177.0 (2)
C7—C11—C12—C13	−3.0 (4)	C20—C19—N4—C25	−1.6 (4)
C11—C12—C13—C5	0.3 (5)	C18—C19—N4—C25	178.2 (2)
C6—C5—C13—C12	3.3 (4)	C20—C19—N4—Zn2	175.5 (2)
C4—C5—C13—C12	−177.2 (3)	C18—C19—N4—Zn2	−4.7 (3)
N3—C14—C15—C16	1.6 (5)	N1—C2—O1—C1	176.7 (2)
C14—C15—C16—C17	−0.4 (5)	C3—C2—O1—C1	−5.4 (4)
C15—C16—C17—C18	−0.7 (4)	N4—C25—O2—C26	177.9 (3)
C15—C16—C17—C22	178.3 (3)	C24—C25—O2—C26	−3.1 (5)
C16—C17—C18—N3	0.9 (4)	C2—N1—Zn1—N2	174.7 (2)
C22—C17—C18—N3	−178.2 (3)	C6—N1—Zn1—N2	−7.20 (16)
C16—C17—C18—C19	180.0 (2)	C2—N1—Zn1—Cl3	61.5 (2)
C22—C17—C18—C19	0.9 (4)	C6—N1—Zn1—Cl3	−120.45 (15)
N3—C18—C19—N4	−3.5 (4)	C2—N1—Zn1—Cl4	−79.8 (2)
C17—C18—C19—N4	177.4 (2)	C6—N1—Zn1—Cl4	98.24 (15)
N3—C18—C19—C20	176.4 (2)	C8—N2—Zn1—N1	−177.6 (3)
C17—C18—C19—C20	−2.7 (4)	C7—N2—Zn1—N1	7.67 (16)
N4—C19—C20—C23	2.3 (4)	C8—N2—Zn1—Cl3	−59.5 (3)
C18—C19—C20—C23	−177.5 (2)	C7—N2—Zn1—Cl3	125.84 (16)
N4—C19—C20—C21	−178.0 (3)	C8—N2—Zn1—Cl4	70.0 (3)
C18—C19—C20—C21	2.1 (4)	C7—N2—Zn1—Cl4	−104.74 (16)
C19—C20—C21—C22	0.5 (5)	C25—N4—Zn2—N3	−175.9 (3)
C23—C20—C21—C22	−179.9 (3)	C19—N4—Zn2—N3	7.41 (18)
C20—C21—C22—C17	−2.4 (5)	C25—N4—Zn2—Cl1	−59.7 (3)
C16—C17—C22—C21	−177.3 (3)	C19—N4—Zn2—Cl1	123.57 (17)
C18—C17—C22—C21	1.7 (5)	C25—N4—Zn2—Cl2	82.5 (2)
C19—C20—C23—C24	−1.0 (4)	C19—N4—Zn2—Cl2	−94.14 (18)
C21—C20—C23—C24	179.3 (3)	C14—N3—Zn2—N4	−179.4 (3)
C20—C23—C24—C25	−0.8 (5)	C18—N3—Zn2—N4	−9.09 (18)
C23—C24—C25—N4	1.6 (5)	C14—N3—Zn2—Cl1	65.8 (3)
C23—C24—C25—O2	−177.3 (3)	C18—N3—Zn2—Cl1	−123.84 (17)
O1—C2—N1—C6	177.4 (2)	C14—N3—Zn2—Cl2	−64.1 (3)
C3—C2—N1—C6	−0.5 (4)	C18—N3—Zn2—Cl2	106.21 (17)

## References

[bb1] Bruker (1997). *SMART* and *SAINT* Bruker AXS Inc., Madison, Wisconsin, USA.

[bb2] Sheldrick, G. M. (1996). *SADABS* University of Göttingen, Germany.

[bb3] Sheldrick, G. M. (2008). *Acta Cryst.* A**64**, 112–122. 10.1107/S0108767307043930 18156677

[bb4] Zheng, S.-L., Zhang, J.-P., Wong, W.-T. & Chen, X.-M. (2003). *J. Am. Chem. Soc.* **125**, 6882–6883. 10.1021/ja029097a 12783537

